# Popular Diabetes Apps and the Impact of Diabetes App Use on Self-Care Behaviour: A Survey Among the Digital Community of Persons With Diabetes on Social Media

**DOI:** 10.3389/fendo.2019.00135

**Published:** 2019-03-01

**Authors:** Mihiretu M. Kebede, Claudia R. Pischke

**Affiliations:** ^1^Leibniz Institute for Prevention Research and Epidemiology-BIPS Bremen, Germany; ^2^Health Sciences, University of Bremen, Bremen, Germany; ^3^College of Medicine and Health Science, Institute of Public Health, University of Gondar, Gondar, Ethiopia; ^4^Medical Faculty, Centre for Health and Society, Institute of Medical Sociology, University of Düsseldorf, Düsseldorf, Germany

**Keywords:** self-care, diabetes applications, diabetes apps, diabetes, type 1 diabetes, type 2 diabetes

## Abstract

**Introduction:** This study aimed to identify popular diabetes applications (apps) and to investigate the association of diabetes app use and other factors with cumulative self-care behaviour.

**Methods:** From November 2017 to March 2018, we conducted a web-based survey with persons 18 years of age and above. We recruited respondents via diabetes Facebook groups, online patient-forums and targeted Facebook advertisements (ads). Data on participants' demographic, clinical, and self-management characteristics, as well as on self-care behaviour and characteristics of the diabetes apps use were collected. Self-care behaviour was measured using a licensed version of the Summary of Diabetes Self-care Activities (SDSCA) questionnaire. The cumulative self-care score was calculated by summing up scores for “general diet,” “specific diet,” “exercise,” “blood glucose testing,” “foot care” and “smoking.” To identify popular diabetes apps, users were requested to list all apps they use for diabetes self-management. Two sample *t*-test and multiple linear regression stratified by type of diabetes were performed to examine associations between app use and self-care behaviour, by controlling for key confounders.

**Results:** One thousand fifty two respondents with type 1 and 630 respondents with type 2 diabetes mellitus (DM) entered the survey. More than half, 549 (52.2%), and one third, 210 (33.3%), of respondents with type 1 and 2 DM, respectively, reported using diabetes apps for self-management. “mySugr” and continuous glucose monitoring apps, such as “Dexcom,” “Freestyle Libre,” and “Xdrip+” were some of the most popular diabetes apps. In both respondent groups, the cumulative self-care behaviour score was significantly higher among diabetes app users (compared to non-users) and scores for three individual self-care components, namely “blood glucose monitoring,” “general diet,” and “physical activity” were significantly higher among diabetes app users than among non-users. After adjusting for confounding factors, diabetes app use increased the cumulative self-care score by 1.08 (95%CI: 0.46–1.7) units among persons with type 1 DM and by 1.18 (95%CI: 0.26–2.09) units among persons with type 2 DM, respectively.

**Conclusion:** For both, persons with type 1 and type 2 diabetes, using diabetes apps for self-management was positively associated with self-care behaviour. Our findings suggest that apps can support changes in lifestyle and glucose monitoring in these populations.

## Introduction

In 2045, the global population affected by diabetes mellitus (DM) is projected to rise from 425 million reported in 2017 (1) to 693 million (2). Diabetes is considered as one of the most challenging health problems of the Twenty first century (2) and remains one of the most expensive diseases (3). About 850 billion USD were spent on the treatment of the disease only in the year 2017 (1). This global diabetes healthcare expenditure is expected to continue growing (4).

Good diabetes management following a standardized medical and behavioural treatment protocol improves quality of life, and may prevent complications and premature mortality (5). In addition to medical treatment, effective interventions promoting healthy behaviour are important aspects of diabetes care (6–8). Regular physical activity, blood glucose monitoring, and optimal adherence to medication and recommendations for a balanced diet are integral to effective diabetes self-management (9, 10). Diabetes self-management is a key determinant of successful and cost-effective diabetes care that markedly reduces hospital admissions as well as complications (11–13). However, diabetes self-management is a highly demanding responsibility which requires continuous diabetes education and support to empower patients in improving health literacy and maintaining the necessary self-care behaviours (14, 15). Evidence suggests that diabetes applications (apps) support patients in advancing their knowledge of the disease, including awareness of complications and their personal self-management capabilities (16–20). Previous studies showed improvements in glycemic controls from digital health interventions including the use of diabetes apps (10, 18, 21–23). Smartphone diabetes apps enable patients to keep track of their physical activity, nutrition, and blood glucose monitoring (24–27). In addition, tailored diabetes self-management interventions and personalized recommendations can be facilitated by diabetes apps (28, 29). Through diabetes apps, patients can monitor their progress towards achieving personal glycemic and behavioural goals (30). The Agency for Health Care Research and Quality reported five diabetes applications (apps) which were effective in reducing glycated hemoglobin levels (HbA1c) (31). Additional apps were shown to support patients in reducing high or low glycemic abnormalities, improving treatment satisfaction, and self-care behaviour (31). Further, the International Diabetes Federation (IDF) recently indicated that “well-suited” diabetes apps might be important for promoting diabetes self-management practices and to prevent complications (32, 33). The rapid progress of internet of things, big data analytics, machine learning, artificial intelligence and other advances in mobile computing (34) are revolutionizing the future of personalized diabetes medicine.

The opportunities availed by diabetes apps have attracted many healthcare stakeholders including providers, payers, consumers and developers. The digital diabetes market is rapidly growing and it is expected to reach a worth of 742 million USD in 2022 (35). In 2017, the R2G(Research to guidance) released a report on mHealth app economics that stated diabetes is the best market for digital health innovation (36). Many diabetes apps are already available on typical app stores.

Multiple intervention studies have investigated the role of diabetes apps in improving self-care behaviour, such as glucose monitoring, diet, foot care, and physical activity in clinical settings (24, 37–40). Evidence on whether diabetes apps improve diabetes self-care behaviour in real world settings is still limited. Moreover, only few studies evaluated the content of diabetes apps available in the popular stores (26, 41–43), and remains unclear to date which are the most frequently used and appraised DM apps. Therefore, this study aimed to identify popular diabetes apps and to investigate the association of diabetes app use and other factors with cumulative self-care behaviour in persons with type 1 and 2 diabetes, applying a social media survey approach.

## Methods

### Study Design, Source of Respondents, and Questionnaire Design

From November, 2017 to March, 2018, we conducted a web-based survey in the online community of persons with diabetes. The design of the web-based survey was adjusted to have computer and smartphone friendly layout options.

We used Facebook groups, targeted Facebook advertisements (ads) and online diabetes patient-forums to recruit respondents. The full detail of the recruitment process is described elsewhere (21). In short, using lime survey (44), a web-based questionnaire was designed in German and English which included questions about diabetes status, demographic characteristics, type of diabetes, medication use, self-care behaviour, blood-glucose level, perceived confidence regarding self-management capacity and perceived metabolic control. In addition, questions about smartphone ownership, type of smartphone owned and diabetes smartphone app use were asked. Self-care behaviour was measured with a licensed version of the Summary of Diabetes Self-care Activities Questionnaire(SDSCA) (45). Questions regarding diabetes smartphone apps were adapted based on questions of the Mobile App Rating Scale (MARS) (8). To identify popular diabetes apps, app users were requested to list all apps they use for diabetes self-management.

To recruit respondents, we used a systematic keyword search on Facebook to identify closed, secret and public diabetes Facebook groups held in English or German. After identification of the Facebook groups, we submitted requests to join each group. The group requests were submitted with messages containing the survey URL and information about the aim of our study. Personal messages were sent to admins and moderators of the Facebook groups to explain the purpose of the survey, ethical aspect of the study, authenticity, and the time required to complete the survey. After receiving approval for the submitted requests, the survey URL accompanied by explanations about informed consent and the time required to complete the survey was posted on each diabetes group's Facebook page to invite group members to anonymously participate in the survey. In addition, we run 10 targeted ads reaching about 30,000 people potentially living with diabetes in German and English speaking countries. The targeted Facebook ads were conducted to address persons who were 18 years and older, living in English (Australia, Canada, United Kingdom and United States) or German speaking countries (Germany, Switzerland and Austria). People living in these countries with an interest in pages containing diabetes-related terms, such as “cure diabetes,” “diabetes health,” and “glycemic index” were targeted. Moreover, we searched diabetes-specific online forums available on Google. To incentivize participation, 10 Amazon vouchers each costing 50 euros were given to participants in a lottery.

### Ethical Standards

The survey adheres with the ethical standards of the Leibniz Institute for Prevention Research and Epidemiology. The University of Bremen Central Research Development Fund committee also approved the study and funded the cost of the Amazon vouchers. Before taking part in the survey, written explanation was provided to inform all respondents about the anonymity of the survey. They were also informed about taking part to the survey is fully voluntarily, their responses will be kept confidential and can skip from answering any question they are not comfortable or stop participating in the survey at any stage. Respondents were also required to electronically give their consent before their taking part in the survey. Participants were asked to provide their email addresses if and only if they want to participate in the 50€ Amazon vouchers. The email addresses were redirected to be stored in a separate database and answers were not linked to any of the email addresses. After, the random selection of the email addresses for providing the incentives for winners, email addresses data were permanently erased.

### Data Management and Statistical Analysis

To warrant the quality of data, the primary investigator checked the responses one by one on a daily basis until the survey period was completed. Multiple responses received from a similar Internet Protocol (IP) address were discarded.

After completion of the survey, data from lime survey were exported to Microsoft Excel. R studio version 3.5.1 statistical software (46) was used to analyze the data. Descriptive statistics and linear regression analyses were conducted. Characteristics of diabetes app use were analyzed using descriptive statistics. To identify popular diabetes apps in both persons with type 1 and 2 diabetes, all the names of the apps listed by each respondent were investigated one by one and counted for each respondent. Frequency of the named diabetes apps were calculated for both types of diabetes.

By following the American Diabetes Association guideline, self-reported glycemic control and HbA1c-level were categorized into hypoglycemia, hyperglycemia and good glycemic control (47, 48). In addition, eight “yes” or “no” questions were asked to measure respondents' concerns regarding their diabetes self-management. The questions were about respondent's concern feeling hypoglycaemia and hyperglycaemia, forgetting to measure blood glucose levels and to take medications, not knowing whom to contact in case of a need for assistance, being left out of medication or supplies and feeling unsure about how to calculate insulin doses. The “yes” and “no” responses for these questions were coded as 1 and 0, respectively. The total score for diabetes self-management concern was calculated for each respondent by adding up all the individual scores of the scale. The total score was then categorized into “low” and “high” concern using a median split (median = 3) after checking for normal distribution using the Shapiro-Wilk test.

Perceived confidence on diabetes self-management was measured by a likert scale question by which the response ranges from “not confident at all” to “very confident.” Similarly, perceived metabolic control was also measured by a likert scale question with responses ranging from “very well-controlled” to “very poorly-controlled.”

The SDSCA includes subscales that measure “general diet,” “specific diet,” “exercise,” “blood glucose testing,” “foot care” and “smoking” over the past week. Scores were created as recommended for the tool (45). Accordingly, the total number of days for each self-care activity was calculated for each respondent. Responses for smoking were recoded as “1” for “non-smokers” and “0” for smokers. Then a cumulative score of self-care was calculated for each respondent by summing up all scores of the individual self-care behaviours. To check whether there was a statistically significant difference between diabetes app users and non-users regarding the scores of the cumulative and individual self-care components, two-sample *t*-tests were performed.

In addition, after checking normality of the self-care data distribution, the association of diabetes app use with the self-care score was analysed using multiple linear regression stratified by type of diabetes. Two linear models were fit for type 1 and type 2 diabetes, respectively. Variables, such as age, sex, educational status, glucose lowering medication use, self-reported rating of metabolic control, perceived confidence in diabetes self-management, diabetes self-management concern, and mobile app use skill and diabetes app use were included a priori in the models.

Regression coefficients with *p* < 5% were considered statistically significant. Models were evaluated by visually examining the linearity of residuals and assumptions underlying multiple linear regression were checked by using appropriate R commands (49). Hence, multiple linear regression such as homoscedasticity of variance were checked by using Breuch Pagan test (“bptest”) from the “lmtest” package (50) and using the “gvlma” packages in R (49). Multicollinearity among the variables was evaluated by checking the correlation matrix of the variables included in the model and by investigating the variance inflation factor of each variable. The effect of multicollinearity was ignored if the correlation value was <0.4 and the variance inflation factor <2.0. There was no evidence of violations of linear model assumptions. In addition, the results of the multicollinearity assessment for both models shows that no variable has a variance inflation factor value of more than 2.0 suggesting multicollinearity among the variables is negligible. Visualization of the data and exportation of the outputs of regression were performed using the Hadley Wickham's “ggplot2” as well as “sjPlot packages in R, respectively (51, 52).

## Results

### Characterstics of Respondents

A total of 1682 complete responses were received from respondents with type 1 or type 2 diabetes who owned a smartphone. Of these, 1,052 (62.6%) were respondents with type 1 DM. The majority of respondents with type 1 diabetes were female 763 (72.5%) and 420 (66.7%) were female and had type 2 DM. The mean age (SD) of the respondents were 39 (*SD* = ±12.9) for DM type 1 and 52.9 (*SD* = ±11.4) years for DM type 2, respectively. Most respondents came from high income countries (see Table 1).

**Table 1:** Characteristics of the respondents.

	**Persons with type 1 DM**	**Persons with type 2 DM**
Age, mean(SD)	39 (12.9)	52.9 (11.4)
≤40	591 (56.2)	99 (15.7)
40-60	400 (38)	346 (54.9)
60+	61 (5.8)	185 (29.4)
**SEX**		
Female	763 (72.5)	420 (66.7)
Male	289 (27.5)	210 (33.3)
**EDUCATIONAL STATUS**		
Primary to secondary	410 (39)	278 (44.1)
Polytechnic diploma	184 (17.5)	117 (18.6)
Bachelor degree and above	458 (43.5)	235 (37.3)
**CONTINENT**		
USA/Canada/Central America	353 (33.6)	276 (43.8)
Europe	607 (55.7)	239 (37.9)
Oceania	52 (4.9)	24 (3.8)
Asia	15 (1.4)	67 (10.6)
Africa and Latin America	25 (2.4)	24 (3.8)
RESPONDENTS' COUNTRY INCOME LEVELS ^*^		
High income	1,012 (96.2)	540 (85.7)
Upper-middle income	30 (2.9)	22 (3.5)
Low to lower-middle income	19 (0.95)	68 (10.8)
Total	1,052 (100)	630 (100)

* *Based on the World Bank 2017–2018 country classifications (53)*.

### Clinical Characteristics and Diabetes Self-Management Experiences of Respondents

More than 95% (1,004) and 86% (541) of respondents with type 1 and type 2 DM reported taking glucose lowering medications, respectively. Nearly one-third of respondents with type 1 and one-fourth with type 2 DM reported that they first consult Facebook groups, diabetes smartphone apps or the internet whenever they have concerns regarding their diabetes self-management. Only approximately two-thirds reported first consulting a diabetes specialist team or other health care providers. Regarding the problems experienced in diabetes self-management, the feelings of symptomatic hyperglycaemia and hypoglycaemia were reported among both, respondents with type 1 and type 2 DM (Table 2).

**Table 2:** Clinical and self-management characteristics of respondents with type 1 and type 2 DM.

	**Persons with type 1 DM N (%)**	**Persons with type 2 DM N (%)**
**ON GLUCOSE LOWERING MEDICATION**		
Yes	1,004 (95.4)	541(85.9)
No	48 (4.6)	89 (14.1)
**IF YOU HAVE CONCERNS REGARDING YOUR DIABETES MANAGEMENT**		
**WHERE DO YOU GO FIRST FOR ASSISTANCE?**		
Diabetes specialist team/healthcare provider	660 (62.7)	431 (68.4)
Facebook group/Internet/smartphone app	316 (30)	153 (24.3)
Support group/friends/family	66 (6.3)	38 (6)
Other	10 (1)	8 (1.3)
**PROBLEMS WITH DIABETES SELF-MANAGEMENT**		
**Feeling symptomatic low blood sugar**		
Yes	663 (63)	121 (19.2)
No	389 (37)	509 (80.8)
**Feeling symptomatic high blood sugar**		
Yes	532 (50.6)	200 (31.8)
No	520 (49.4)	430 (68.2)
**Forgetting to measure blood sugar levels**		
Yes	247 (23.4)	175 (22.8)
No	805 (76.5)	455 (72.2)
**Forgetting to take medication or insulin**		
Yes	186 (17.7)	109 (17.3)
No	866 (82.3)	521 (82.7)
**Not knowing how to identify high or low blood sugars**		
Yes	57 (5.4)	65 (10.3)
No	995 (94.6)	565 (89.7)
**Not knowing whom to contact when in need of assistance**		
Yes	41 (3.9)	50 (7.9)
No	1,011 (96.1)	580 (92.1)
**Being left without medication/supplies**		
Yes	105 (10)	44 (7)
No	947 (90)	586 (93)
**Felt unsure about how to calculate your insulin/glucose lowering**		
**medication dose**		
Yes	187 (17.8)	34 (5.4)
No	865 (82.2)	596 (94.6)
**Diabetes self-management concern**		
High concern	637 (39.3)	411 (65.2)
Low concern	415 (60.7)	219 (34.8)
**Diabetes app use**		
Yes	549 (52.2)	210 (33.3)
No	503 (47.8)	420 (66.7)
**Use CGM**		
Yes	296 (28.1)	218 (3.3)
No	756 (71.9)	609 (96.7)
**Perceived metabolic control**		
Well controlled	655 (62.4)	323 (51)
Neutral	256 (24.4)	154 (25)
Poorly controlled	139 (13.2)	151 (24)
**Self-reported confidence on diabetes self-management**		
Very confident	706 (67.2)	282 (44.8)
Neutral	122 (11.6)	97 (15.4)
Not confident at all	222 (21.1)	251 (39.8)
Total	1,052 (100)	630 (100)

### Characteristics of Diabetes App Use

More than half, 572 (54.5%) respondents with type 1 and more than two third, 432 (68.8%), with type 2 DM reported owning an Android smartphone. The majority of the respondents with type 1 DM, 572 (54.5%) reported being highly skilled or experts in installing and using a mobile app. Of those who were currently using diabetes apps for their self-management, 120 (21.9%) of respondents with type 1 diabetes reported using their app for calculating insulin doses, of which 29 (25%) mentioned that they had erroneous results in calculating insulin doses with these apps. The most commonly used app functionality were using them as diaries for blood glucose and for tracking meal and carbohydrate intakes. The majority of the respondents with type 1 (58.4%) and type 2 (65.4%) reported that their diabetes app was perfectly easy to navigate (Table 3).

**Table 3:** Distribution of characteristics of diabetes app use by diabetes type.

**Choice**	**Persons with type 1 DM, N (%)**	**Persons with type 2 DM, N (%)**
**Type of smartphone**	572 (54.5)	432 (68.8)
Android	458 (43.7)	174 (27.7)
Apple (iPhone)	13 (1.7)	172.7
Windows	6 (0.6)	5 (0.8)
**APP INSTALLING/USING SKILL**		
Highly skilled or expert user	560 (53.4)	232 (37)
Good skill	436 (41.6)	309 (49.4)
Poor skill	53 (5)	85 (13.6)
**INSTALLED DIABETES APP**		
No	503 (47.8)	420 (63.7)
Yes	549 (52.2)	210 (33.3)
	1,052	630
**Problems encountered with the diabetes apps**	*N* = 549	*N* = 210
Crashing of software	66 (12)	13 (6.2)
Difficulty of understanding advice given by the app	32 (5.8)	9 (4.3)
Results that do not align with other medical advise you have been given	33 (4.3)	12 (5.7)
No problems	395 (72)	161 (76.7)
**USE YOUR APP TO CALCULATE YOUR INSULIN DOSE**		
Yes	120 (21.9)	17 (8)
No	429 (78.1)	193 (92)
**Had problems with insulin does calculator**	*N* = 120	*N* = 17
Wrong insulin dose calculations	22 (18.3)	0
Insulin dose provided without entering the necessary values	7 (5.8)	0
**USEFUL FEATURES OF THE APPS: MULTIPLE RESPONSES**		
Diary of blood glucose levels	423 (77)	184 (91.4)
Reminders to check blood glucose levels	131 (23.9)	47 (22.4)
Diary of meals and carbohydrate intake	279 (52.5)	106 (50.5)
Calculation device to determine insulin dose	134 (24.2)	24 (11.4)
Guidelines of ideal blood glucose measurements	116 (21.1)	57 (27.1)
Calendar of diabetes related appointments	62 (11.3)	22 (10.5)
Contact details for your diabetes team or General practitioner	64 (11.8)	27 (12.8)
Dietary advice	85 (15.5)	40 (19)
Your contact details and condition information	55 (10)	30 (14.3)
**FREQUENCY OF APP USE**		
Never	26 (4.7)	7 (3.3)
Only when needing guidance	52 (9.5)	7 (3.3)
Monthly	17 (3.1)	3 (1.4)
Weekly	21 (3.8)	12 (5.7)
Few days per week	44 (8)	26 (12.4)
Daily	168 (30.7)	60 (28.6)
Every time a person eats or takes	220 (40.2)	95 (45)
**USEFULNESS OF THE DIABETES APP**		
Not at all useful	10 (1.8)	5 (2.4)
Not very useful	17 (3.1)	11 (5.3)
Somewhat useful	143 (26)	62 (29.7)
Very useful	184 (33.6)	76 (36.4)
Extremely useful	194 (35)	55 (26.3)
**HOW WELL DOES THE DIABETES APP FUNCTION**		
Does not function	5 (0.9)	4 (1.9)
Some functions work, but slow or has technical problems	14 (2.6)	7 (3.4)
App works overall, but slow or has technical problems at times	46 (8.4)	19 (9.2)
Mostly functional with minor problems	275 (50.5)	55 (26.6)
Perfect with no technical problems	205 (37.6)	122 (58.9)
**HOW EASY IS IT TO LEARN HOW TO USE THE DIABETES APP**		
There are no/limited user instructions, or it is confusing	7 (1.3)	7 (3.3)
Useable after a lot of time/effort	21 (3.8)	4 (1.9)
Useable after some time/effort	75 (13.7)	23 (10.9)
Easy to learn to use with given instructions	186 (34)	69 (32.9)
Able to use immediately, simple	258 (47.2)	107 (51)
**HOW EASY IS IT TO NAVIGATE THROUGH YOUR APP?**		
Different sections within the App are disconnected	9 (1.7)	7 (3.3)
Easy after a lot of time/effort	16 (2.9)	6 (2.9)
Easy after some time/effort	98 (18)	32 (15.4)
Easy but missing minor links	104 (19)	27 (13)
Perfectly easy	319 (58.4)	136 (65.4)
**HOW DO YOU FIND THE LAYOUT/DESIGN OF YOUR APP?**		
Very poor, some options are impossible to locate	6 (1.1)	4 (1.9)
Poor, some options are difficult to locate	24 (4.4)	10 (4.8)
Satisfactory, few problems with selecting options	119 (21.8)	37 (17.9)
Good, able to locate all options	240 (44)	96 (46.4)
Excellent, logical and clear layout	156 (28.6)	60 (29)
**REASONS FOR NOT USING AN APP**		
Didn't know they existed	83 (16.8)	175 (42.9)
They do not work on my mobile phone	32 (6.5)	11 (2.7)
Cost	24 (4.9)	29 (7.1)
Feel confident without one	111 (22.5)	77 (18.9)
Have tried one before and didn't like it	156 (31.6)	63 (15.44)
**WOULD YOU BE INTERESTED IN USING A SMARTPHONE APP TO ASSIST WITH YOUR DIABETES MANAGEMENT?**		
Yes	310 (61.6)	218 (51.9)
No	193 (38.4)	202 (48.1)

**Figure 1:**
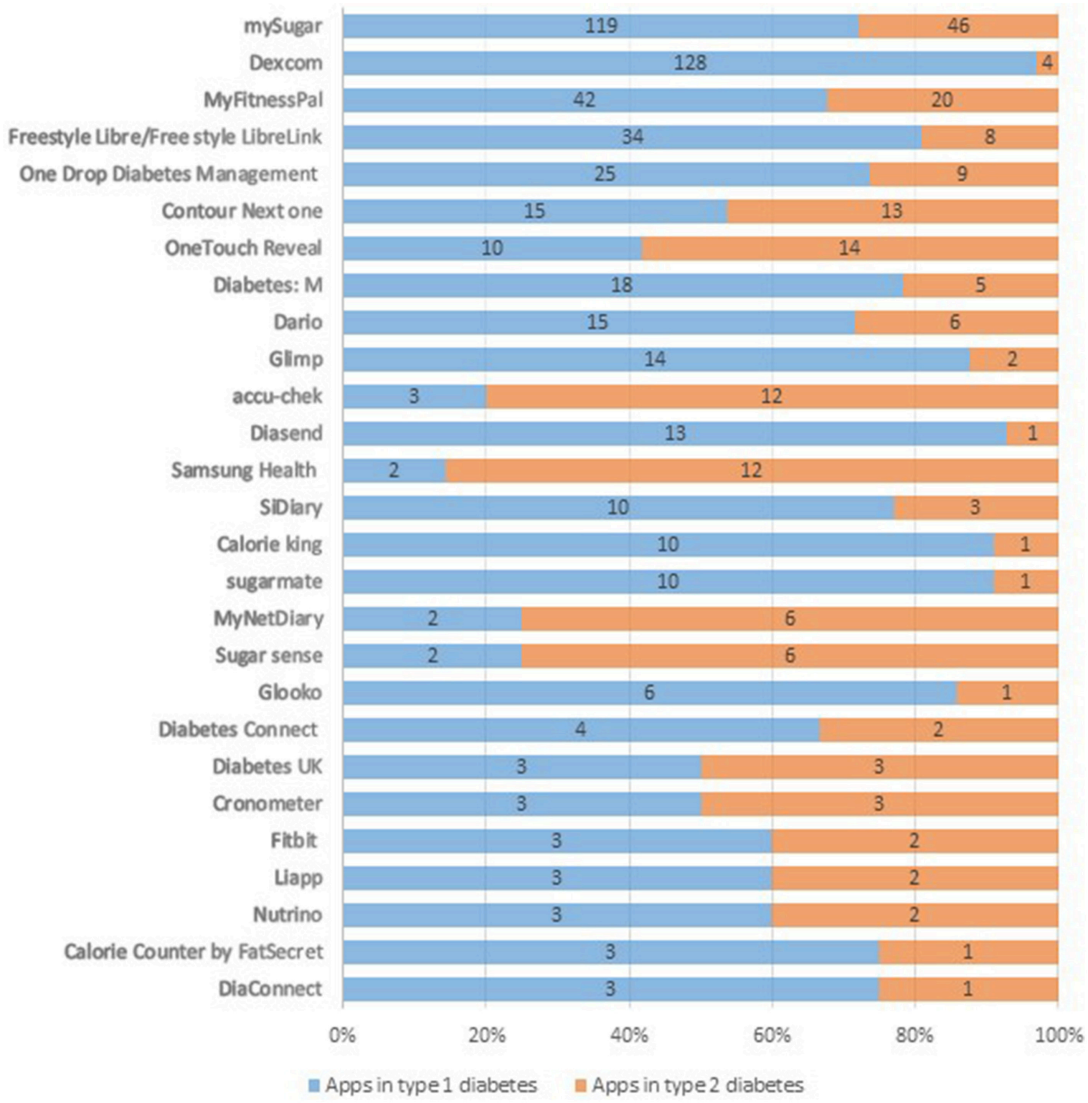
Popular diabetes apps among persons with type 1 and type 2 DM (names of the apps and their frequency presented in absolute counts). Percentages labels on the x-axis represent proportions of a specific diabetes app reported by respondents with type 1 and 2 DM from the total users of that app.

Overall, 145 different diabetes apps were reported by respondents. A detailed list of all reported diabetes apps is available in the Supplementary Material. The app “mySugr” was the most popular app reported by 165 of the 759 of respondents who reported using apps for diabetes self-management. Continuous glucose monitoring apps such as “Dexcom,” “Freestyle Libre” and “Xdrip+” were the most popular diabetes apps among respondents with type 1 DM (Figure 1).

### Association of Diabetes App Use With Self-Care Behaviour Among Persons With Type 1 and Type 2 DM

**Figure 2:**
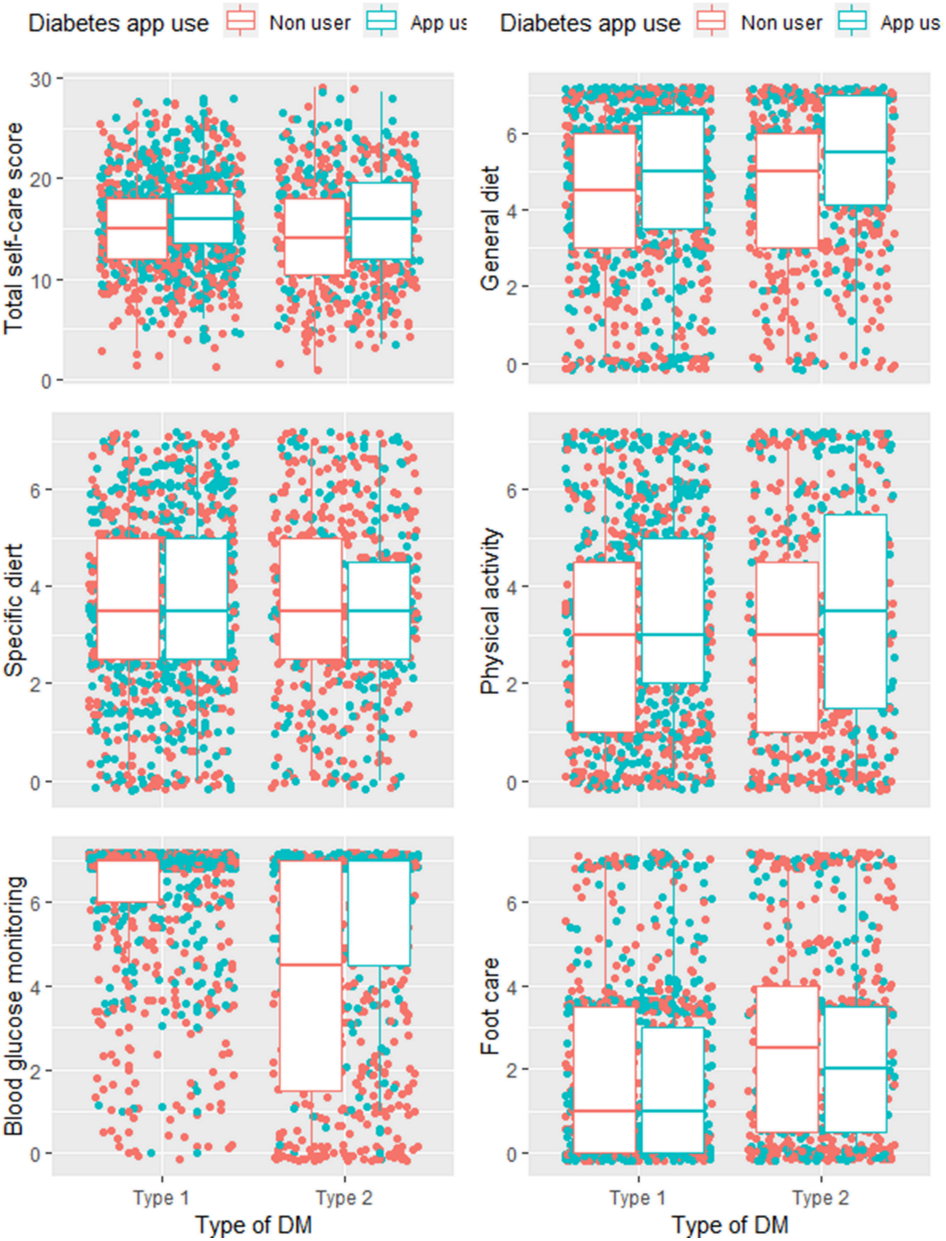
Distribution of individual and total self-care scores for diabetes app users and non-users with type 1 and type 2 DM.

Figure 2 displays the distribution of the self-care scores for different components comparing app users and non-users, stratified by DM type. For both, persons with type 1 and type 2 DM, the total scores of almost all self-care components and the cumulative self-care score were higher among diabetes app users. The difference is larger for both groups of respondents in two self-care behaviours: “general diet” and “physical activity” (Figure 2).

The cumulative self-care score, as well as the individual self-care components, except for foot care and specific diet were significantly higher among diabetes app users, both with type 1 and type 2 DM (Table 4).

**Table 4:** Self-care behaviour differences among diabetes app users and non-users.

**Self-care behaviour**	**Type 1 diabetes**			**Type 2 diabetes**		
	**Diabetes app non-users mean(SD)**	**Diabetes app users mean(SD)**	**Difference (*p*-value)**	**Diabetes app non-users mean(SD)**	**Diabetes app users mean(SD)**	**Difference (*p*-value)**
General diet (aggregate)	4.34 (2.08)	4.75 (1.93)	0.000^*^	4.44 (1.93)	5.2 (1.79)	0.000^*^
Specific diet (aggregate)	3.54 (1.83)	3.56 (1.74)	0.86	3.63 (1.8)	3.54 (1.85)	0.564
Physical activity (aggregate)	2.93 (207)	3.43 (2.09)	0.0001^*^	2.91 (2.18)	3.46 (2.38)	0.006^*^
Blood Glucose Monitoring	6.03 (1.8)	6.63 (1.0)	0.0000^*^	4.06 (2.7)	5.71 (1.92)	0.000^*^
Foot care(aggregate)	1.86 (2.07)	1.67 (2.02)	0.132	2.66 (2.41)	2.48 (2.32)	0.358
Cumulative self-care score	15.1 (4.82)	16.1 (4.15)	0.000^*^	14.1 (5.37)	16.0 (5.33)	0.000^*^

* *statistically significant*.

### Factors Associated With Self-Care Behaviour Among Persons With Type 1 DM

**Figure 3:**
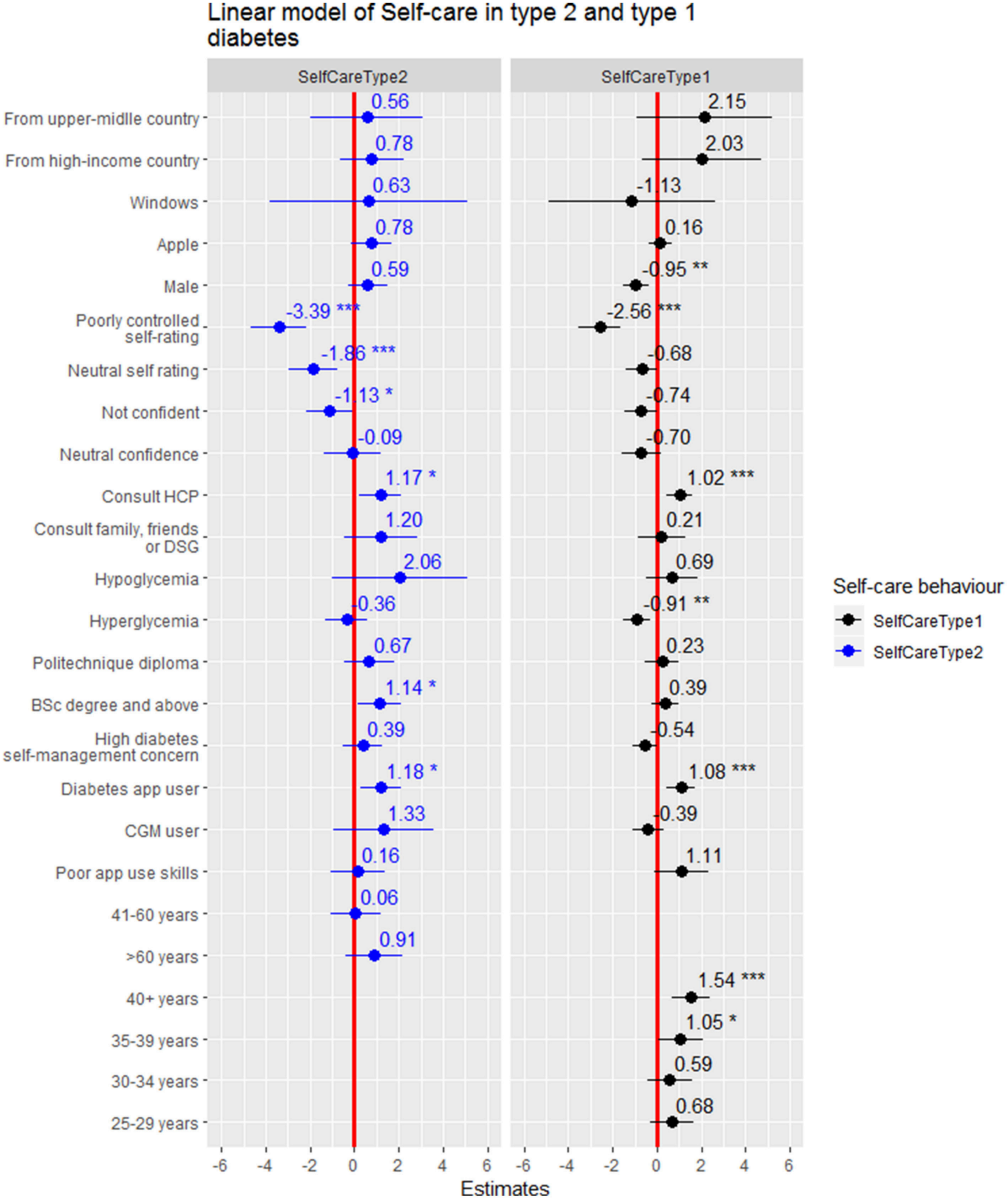
Forest plot of the coefficients with 95%CI for factors of self-care behaviour in persons with type 1 vs. type 2 DM. ^*^statistical significant at *p* < 0.05, ^**^statistical significant at *p* < 0.005, ^***^statistical significant at *p* < 0.001.

In persons with type 1 DM, using diabetes apps for self-management, being older, consulting diabetes specialist teams or other health care providers were positively associated with higher self-care behaviour scores. However, male sex, having hyperglycaemia, and having a self-rated “poorly-controlled” metabolic control were significantly associated with lower self-care behaviour (Figure 3). Using diabetes apps for self-management increased self-care by 1.08 (95%CI: 0.46–1.7) units. Self-care behaviour among respondents with type 1 diabetes increased by 1.05 (95%CI: 0.04–2.07) and 1.54 (95%CI: 0.70–2.39) units among those respondents who were 35–39 years and older than 40 years of age, respectively than respondents who were between 18 and 24 years old. Respondents who consulted diabetes specialist team or health care provider had 1.02 units of higher self-care behaviour compared to respondents who first consult Facebook groups/smartphone apps/internet for assistance (Table 5).

**Table 5:** Factors associated with self-care behaviour in persons with type 1 and type 2 diabetes.

	**Linear model for type 1 DM model**			**Linear model for type 2 DM model**		
**Predictors**	**Estimates**	**Conf. Int (95%)**	***p***	**Estimates**	**Conf. Int (95%)**	***p***
Intercept	12.69	9.84 to 15.54	<0.001^*^	13.13	11.10 to 15.17	<0.001^*^
**AGE GROUP**						
18–24	Ref	Ref	Ref			
25–29 years	0.68	−0.32 to 1.68	0.181			
30–34 years	0.59	−0.41 to 1.58	0.249			
35–39 years	1.05	0.04 to 2.07	0.042^*^			
40 + years	1.54	0.70 to 2.39	<0.001^*^			
≤40 years				Ref	Ref	Ref
41–60 years				0.06	−1.09 to 1.20	0.923
>60 years				0.91	−0.38 to 2.20	0.168
**EDUCATIONAL STATUS**						
Politechnique diploma	0.23	−0.53 to 0.99	0.550	0.67	−0.45 to 1.79	0.242
Bachelor degree and above	0.39	−0.22 to 1.00	0.211	1.14	0.16 to 2.12	0.022^*^
**SEX**						
Female	Ref	Ref	Ref	Ref	Ref	Ref
Male	−0.95	−1.54 to −0.36	0.002^*^	0.59	−0.30 to 1.47	0.193
**FIRST CONTACT FOR ASSISTANCE**						
Facebook group/Internet/Smartphone app	ref	Ref	Ref	Ref	Ref	Ref
Health care provider	1.02	0.44 to 1.60	0.001^*^	1.17	0.22 to 2.12	0.015^*^
Friends, family or DSG	0.21	−0.87 to 1.29	0.702	1.20	−0.46 to 2.87	0.155
**RESPONDENTS'ORIGIN**						
From low-income country	Ref	Ref	Ref		Ref	Ref
Upper-midlle country	2.15	−0.90 to 5.21	0.167	0.56	−2.00 to 3.11	0.668
High-income country	2.03	−0.65 to 4.71	0.138	0.78	−0.66 to 2.22	0.287
**TYPE OF SMARTPHONE**						
Android	Ref	Ref		Ref	Ref	Ref
Apple	0.16	−0.38 to 0.69	0.563	0.78	−0.14 to 1.71	0.097
Windows	−1.13	−4.90 to 2.63	0.555	0.63	−3.84 to 5.09	0.782
**APP USE SKILLS**						
Highly skilled	Ref	Ref	Ref	Ref	Ref	Ref
Poorrly skilled	1.11	−0.12 to 2.34	0.077	0.16	−1.09 to 1.41	0.800
**DIABETES APP USE**						
Non-user	Ref	Ref	Ref	Ref	Ref	Ref
User	1.08	0.46 to 1.70	0.001^*^	1.18	0.26 to 2.09	0.012^*^
**DIABETES SELF-MANAGEMENT CONCERN**						
Low	ref	ref	ref	ref	Ref	ref
High	−0.54	−1.10 to 0.03	0.062	0.39	−0.49 to 1.27	0.383
**CGM USER**						
No	Ref	Ref	Ref	Ref	Ref	Ref
Yes	−0.39	−1.08 to 0.29	0.260	1.33	−0.93 to 3.59	0.248
**GLYCEMIC CONTROL**						
Good	Ref	Ref	Ref	Ref	Ref	Ref
Hyperglycemia	−0.91	−1.54 to −0.27	0.005^*^	−0.36	−1.32 to 0.61	0.467
Hypoglycemia	0.69	−0.49 to 1.86	0.253	2.06	−0.99 to 5.10	0.185
**PERCEIVED METABOLIC CONTROL**						
Well-controlled	Ref	Ref	Ref	Ref	Ref	Ref
Neutral	−0.68	−1.39 to 0.03	0.062	−1.86	−2.95 to −0.77	0.001^*^
Poorly-controlled	−2.56	−3.51 to −1.61	<0.001^*^	−3.39	−4.65 to −2.14	<0.001^*^
**PERCEIVED CONFIDENCE IN DIABETES SELF-MANAGEMENT**						
Highly confident	Ref	Ref	Ref	Ref	Ref	Ref
Neutral	−0.70	−1.61 to 0.21	0.131	−0.09	−1.36 to 1.18	0.889
Not confident	−0.74	−1.49 to 0.01	0.054	−1.13	−2.17 to −0.09	0.034^*^

* *statistically significant*.

However, being male, having hyperglycaemia and having a self-rated “poorly-controlled” metabolic control significantly reduced self-care behaviour by −0.95 (95%CI: −1.54 to −0.36), −0.91 (95%CI: −1.54 to −0.27) and −2.56(95%CI: −3.51 to −1.61) units, respectively (Table 5).

### Factors Associated With Self-Care Behaviour Among Persons With Type 2 DM

Using diabetes apps for self-management, educational status, consulting diabetes specialist teams or other health care providers for assistance and were positively associated with self-care behaviour among respondents with type 2 DM. Conversely, having neutral or poorly-controlled self-rated metabolic control and not feeling confident with regard to diabetes self-management were negatively associated with self-care behaviour (Figure 3). Using diabetes apps, having bachelor's degree and above, consulting diabetes specialist teams or other health-care provider for assistance in dealing with self-management concerns, and increased self-care behaviour by 1.18 (95%CI: 0.26–2.09), 1.14 (95%CI: 0.16–2.12), and 1.17 (95%CI: 0.22–2.12) units, respectively. However, respondents who rated their perceived metabolic control as neutral or poorly-controlled also reported reductions in self-care behaviour by −1.86 (95%CI: −2.95 to −0.77) and −3.36 (95%CI: −4.65 to −2.14) units, respectively. In addition, self-care behaviour among respondents who were not confident in their diabetes self-management was reduced by −1.13(95%CI: −2.17 to −0.09) units compared to respondents who felt highly confident (Table 5).

## Discussion

This study revealed that “mySugr” was the most popular diabetes app in both groups of respondents with type 1 and type 2 diabetes. Continuous glucose monitoring apps were particularly popular apps among respondents with type 1 DM. Compared to those who did not use diabetes apps, those who did had significantly higher cumulative self-care scores, independent of key confounding variables such a age, sex and educational status. Results were similar in respondents with type 1 and type 2 diabetes. Both type 1 and type 2 diabetes respondent groups reported that keeping track of blood glucose levels and keeping a diary of dietary intakes were the most useful features of these apps. A study on popular glucose tracking apps on android and apple store identified 20 most popular apps and reported glucose tracking and physical activity as the most common features of the apps (26). Similarly, Boyle and colleagues reported that recording blood glucose levels was the most favoured functionality in diabetes apps (54). This is due to the fact that glucose tracking is the top priority of diabetes self-management (55). However, whether the apps include additional contents designed according to the Association of American Diabetes Educators' evidence-based self-care recommendations, such as “problem solving,” “reducing risks,” or “healthy coping” requires further exploration of the features of the apps.

In this study, the cumulative and individual scores for self-care behaviour, except for “foot care” and “specific diet” were significantly higher among diabetes app users (compared to non-users), both in respondents with type 1 and type 2 diabetes. These results were confirmed by the findings obtained in the linear models which indicated the significant association of diabetes app use with improved self-care behaviour. The majority of previous studies indicate that app use was significantly related to improving blood glucose monitoring. This might be due to the fact that most apps are mainly designed to support blood glucose monitoring. Randomized control trials and observational studies have shown that using diabetes apps for self-management significantly improves scores of cumulative (40, 56) or individual self-care components (39, 57–59). Blood glucose monitoring (56–59), physical activity (57, 59), general diet (39, 56), specific diet (57–59), and foot care (39, 58) behaviours have been reported to be significantly improved by using diabetes apps. This is mainly due to the reason that diabetes app use may indeed be a useful approach to improve diabetes knowledge, self-management skills, and knowledge about complications which may ultimately enhance self-care practices (60).

For both groups of patients examined in our study, primarily consulting diabetes specialist teams or other health-care providers for assistance to deal with self-management concerns was positively associated with improved self-care behaviour, compared to consulting Facebook groups or internet. Previous studies found that diabetes specialist teams are central to addressing patients' self-care challenges, timely responding to complications and enhancing patient's self-management confidence which ultimately improves the ability to complete self-care tasks (61, 62). Consistent with other studies (63, 64), findings of our study also indicate that having a self-rated poor metabolic control appears to be associated with reduced self-care behaviour in both persons with type 1 and 2 DM.

Not feeling confident regarding diabetes self-management capacity was significantly associated with lower self-care behaviour among respondents with type 2 DM, whereas, higher levels of education was positively associated with increased self-care behaviour. Similar to our findings, another study reported that patients who felt confident regarding self-management experienced less difficulties in completing self-care tasks (65).

Moreover, in our study older age was positively associated with improved self-care behaviour in persons with type 1 DM. In comparison, the evidence demonstrating the association of increasing age with self-care is mixed. Similar to our study, previous studies reported older age-groups to be positively associated with improved self-care behaviour (66, 67), while ability to perform self-care tasks was also found to deteriorate in frail older adults (68, 69). The association of older age and improved self-care behaviour in persons with type 1 DM is partly due to the duration of the disease. Because the onset of the disease occurs at a relatively young age, older age groups with type 1 DM have cultivated self-management knowledge and experience which may enrich the completion of self-care tasks (67). Using apps in this group may therefore just be an expression of high patient competence.

Being male and experiencing hyperglycaemia were negatively associated with self-care in persons with type 1 DM. In line with this finding, a study from Australia reported that men had significantly lower composite self-care scores (67). Findings of studies conducted in the United States and Canada examining gender disparities in self-care behaviour indicated that women reported higher levels of fruit and vegetable consumption, blood glucose testing and foot care than men (70, 71). Causes of gender differences in self-care behaviour needs further research.

In this study, although we looked at the differences between app users and non-users regarding the individual self-care components, we did not examine the factors for each individual self-care component. More research to understand the impact of predictors in addition to diabetes app use is necessary.

## Limitations

The limitations of our study include the fact that all results are based on the data obtained by a web-based survey. The respondents of the survey were recruited via diabetes-specific Facebook groups, targeted advertisements and online forums. As a result, only respondents presumably with high health and digital literacy might have participated in the study which may not reflect self-care behaviour in the general population of persons with diabetes. Interpretation of the results should also take into account self-selection. Psychometric properties of the diabetes self-management concern questions were not also investigated. The question on glucose lowering medication is also too broad to capture difference for those on insulin or other medications unique to type 2 diabetes. Due to the cross-sectional nature of the study, causal relationships cannot be determined. It might be possible those with higher self-care behaviour are more motivated to use diabetes apps. Respondents in our study came from multiple countries, although the majority of them were from high-income German and English speaking countries. For this reason, there is unobserved variation introduced by the diversity of the respondents. This variation may be due to the difference in unobserved individual and population-level characteristics such as sociocultural and healthcare system differences across countries. This variation was not captured in our study. However, considering the significant growth of Facebook use by older adults (72) and looking at the emerging role of social media connectivity in chronic disease self-management education and health promotion (73, 74), persons with diabetes on social media constitute an important and growing population. In this line, a recent study reported that nearly 90% of older adults reported using Facebook and Twitter to find health information (75). In light of this, our research identified popular diabetes apps and investigated the association of diabetes app use with self-care behaviour. However, more research with larger samples is needed to confirm these findings. Using social media for surveying patient groups relies on self-report, and validation especially in a geographically highly diverse sample is a challenge.

## Conclusion

From all reported diabetes apps, “mySugr” and continuous glucose monitoring apps such as “Dexcom,” “Freestyle Libre,” and “Xdrip+” were few of the most popular diabetes apps. After adjusting for the effects of confounders, using diabetes apps for self-management was positively associated with higher self-care behaviour in both types of diabetes. The findings indicate diabetes apps have the potential to augment diabetes self-management and to develop healthier life style. Considering to prescribe a well-suited diabetes app may be important. Future research on diabetes care should include information on app use as it may become an even more important care-moderating factor.

## Data Availability

The datasets collected, used analysed for study can be obtained from the corresponding author on a reasonable request. The codes written for fitting the regression models, graphs and evaluation of the models are publicly available (https://gitlab.com/Mihiretu/diaapprcodes/blob/master/Self_care%20linear%20model.R).

## Author Contributions

MK conceptualized and designed the study, searched the Facebook groups and online forums search, conducted the targeted advertisements, collected the data, performed the data analysis and wrote the manuscript. CP participated in the conception of the study, contributed to the survey design, and critically revised the manuscript.

### Conflict of Interest Statement

The authors declare that the research was conducted in the absence of any commercial or financial relationships that could be construed as a potential conflict of interest.
