# Corrigendum: Popular Diabetes Apps and the Impact of Diabetes App Use on Self-Care Behaviour: A Survey Among the Digital Community of Persons With Diabetes on Social Media

**DOI:** 10.3389/fendo.2019.00220

**Published:** 2019-04-05

**Authors:** Mihiretu M. Kebede, Claudia R. Pischke

**Affiliations:** ^1^Leibniz Institute for Prevention Research and Epidemiology-BIPS, Bremen, Germany; ^2^Health Sciences, University of Bremen, Bremen, Germany; ^3^College of Medicine and Health Science, Institute of Public Health, University of Gondar, Gondar, Ethiopia; ^4^Medical Faculty, Centre for Health and Society, Institute of Medical Sociology, University of Düsseldorf, Düsseldorf, Germany

**Keywords:** self-care, diabetes applications, diabetes apps, diabetes, type 1 diabetes, type 2 diabetes

In the original article, there was an error. The diabetes app “mySugr” was misspelled as “mySugar” throughout the original article.

A correction has been made throughout the article to correct this spelling error.

The authors apologize for this error and state that this does not change the scientific conclusions of the article in any way. The original article has been updated.

